# Prices for Surgical Dressings

**Published:** 1910-06-04

**Authors:** 


### Institutional Letter-Box.

### PRICES FOR SURGICAL DRESSINGS.

The articles "Hospital Contracts and Prices," being of great practical value, are exciting increasing interest week by week. We have received the following letter, and have pleasure in publishing it, together with the reply of the writer of the articles, who is a gentleman of great experience and knows the subject practically and thoroughly from end to end:—

